# Medical Teaching Institutions Reforms Act: Facts and Fictions

**DOI:** 10.12669/pjms.37.5.4551

**Published:** 2021

**Authors:** Zahid Kamal, Muhammad Aleem, Nauman Aziz, Hafiz Muhammad Amjad

**Affiliations:** 1 Zahid Kamal; 2 Muhammad Aleem; 3 Nauman Aziz; 4 Hafiz Muhammad Amjad

Healthcare delivery is a huge, multifaceted and complicated system with a high-stake product- the health. For improving and upgrading any system, a constant process of change is required which is also time-consuming.

*Har Nayi Tameer Ko Lazim Hai Takhreeb-E-Tamam*

*Hai Issi Mein Mushkilat-E-Zindagani Ki Kushood 1*

*(Every new construction requires complete destruction; that is the key to unlock difficulties in life)*

The term “Global Reforms” is used when the entire system is overhauled. The USA administration having one of the biggest healthcare systems in the world, implements major reforms every 25-50 years.^2^ Similarly health delivery systems in the UK^3^ and Australia^4^ are changing continuously. India is our biggest neighbor and facing similar challenges has implemented changes.^5^ Iran has improved its National healthcare system a great deal by involving all the stakeholders.^6^ These are just a few examples that we may see in health care reforms in the world and especially South-East Asia.

Currently, the healthcare system is too much centralized in Pakistan. Punjab, though the most populated (110 Million people) province, is still run from a single secretariat based in Lahore. It leads not only to delay in decision-making but also opens holes for pilferage.^7^ Sometimes the process for purchase of electro-medical equipment takes many months due to a slow functioning system and lack of true autonomy. It may result in a price hike, alteration in the foreign exchange rate and undue litigation.

Implementation of Autonomy in specialized healthcare has been attempted in the past^7^. Earlier in 1998 (Ordinance), 2002 and 2003 in the form of PM&HI Acts (Punjab Medical & Health Institution), but it was not well received by Health professionals and society. The current government started healthcare reforms from the Khyber Pakhtunkhwa (KP) province and promulgated Medical Teaching Institution (MTI) Reforms Act in 2015.^8^ It led to widespread protests by the healthcare providers, mostly due to communication gap and unexplained ambiguities. It has introduced some new concepts to Pakistani society, such as third party audit of the hospital to improve performance.^9^ The Punjab MTI Reforms Act was approved by the Punjab Assembly in 2020. It comprises four main bodies, namely the Search & Nomination Committee, Provincial Policy Board, Board of Governors and Management Committee.^10^ It would invite all the regular employees of the Specialized Health and Medical Education (SH&ME) department to join the MTI on a contract within 60 days. Those who do not want to join would be sent back to the administrative department of the Govt. of Punjab and can be posted elsewhere. They may come back to the same MTI on deputation with mutual consent.

A good way to implement the reforms is by increasing its friends and reducing its foes. There are several potential threats in this system, making the user wary of it; like the risk of increased politicization, favoritism, covert personal agendas of the members and risk of deprivation of common man from affordable healthcare. Some of the issues in this system have been addressed by embedding supports such as the KP government has supported poor masses through *Sehat Card (a healthcare insurance system)*.^11^ Further debating, one of the potential concerns in this Act is the possibility of appointment of a junior doctor to the Director position that can result in disruption in hierarchy.^12^

According to Verhoest et al.^13^ autonomy in management is at its peak when the Board of Governors (BOG) is free from government influence which is the case in the MTI Act. Moreover, the managers can then make and execute policies according to the need of that particular organization. Suhail & Steen conducted a qualitative study in the KP & Punjab regarding Human Resource (HR) autonomy.^14^ They conducted 70 semi-structured interviews of different managerial levels at three large public hospitals in Pakistan. They concluded that decentralization reduces the difference between intended and implemented HR practices. BoG would improve the performance of the health service by close monitoring.^15^ Those who choose to do Private Practice within MTI would get more enticements. Moreover, the presence of senior specialists in the evening would increase overall patient care and discipline. The grant of a single line budget with strict audit would reduce the bureaucratic delays in matters of budget allocation and re-appropriation. They reported that in the KP after MTI Act implementation employees do not have to wait for a vacant position for promotion if they fulfil the criteria. However, they acknowledged the difficulties faced by a KP Hospital in managing two different categories of HR, that is, civil servants and MTI employees. This issue however will resolve in the next few years as the health department has been directed to adjust all the civil servants back into their system thus leaving the MTI hospitals.

According to Suhail & Steen^14^ currently in Punjab the administrators are mere disseminators of information from the Department of Health. The Performance Evaluation Reports (PER/ACR) is just a formality with no impact on the actual working of the employees. Pursuing approval for trivial things and following government directives all the time limit the creativity of local administrators.

Initially, the Punjab Government was planning to implement MTI Reforms Act at twenty-three Teaching Hospitals attached to the five government medical universities. Later, the decision was reviewed because of some reservations shown by the professionals. Moreover, Punjab Government wanted to test it by implementing MTI reforms initially in few hospitals. At the moment it is only implemented at Khawaja M. Safdar Medical College Sialkot.^15^ However, the staff of KMSMC is still not willing to accept its implementation. The Grand Health Alliance (*a body representing doctors, nurses & Paramedics*) stresses that the BOG should include members from its fraternity so the decisions can be made in consensus and also that such member have better understanding of this system than the non-medical persons.

We know that several previous healthcare systems have not been that successful in Pakistan. All the stakeholders should sit together with an open mind and negotiate whatever is primarily in the best interest of patients and the system. This can be taken as an opportunity for filling the space that politicians and bureaucracy would leave for directors and doctors of the hospitals. This system should be implemented with modifications because it will help the public hospitals to become cost effective, respond quickly, manage resources efficiently and offer their services meritoriously.

## Authors Contribution:

All the authors equally contributed to the study.
